# Primary resistance to first-generation EGFR-TKIs induced by *MDM2* amplification in NSCLC

**DOI:** 10.1186/s10020-020-00193-z

**Published:** 2020-07-01

**Authors:** Dantong Sun, Yan Zhu, Jingjuan Zhu, Junyan Tao, Xiaojuan Wei, Yang Wo, Helei Hou

**Affiliations:** 1 Precision Medicine Center of Oncology, The Affiliated Hospital of Qingdao University, 16 Jiangsu Road, Qingdao, 266000 Shandong China; 2 Department of Medical Oncology, The Municipal Hospital of Qingdao, Qingdao, 266000 Shandong China; 3 Department of Radiation Oncology, The Affiliated Hospital of Qingdao University, Qingdao, 266000 Shandong China; 4 Department of Thoracic Surgery, The Affiliated Hospital of Qingdao University, Qingdao, 266000 Shandong China

**Keywords:** *MDM2* amplification, Primary resistance, EGFR-TKIs, NSCLC, Prognosis

## Abstract

**Introduction:**

Targeted therapy for NSCLC is rapidly evolving. EGFR-TKIs benefit NSCLC patients with sensitive EGFR mutations and significantly prolong survival. However, 20–30% of patients demonstrate primary resistance to EGFR-TKIs, which leads to the failure of EGFR-TKI treatment. The mechanisms of primary resistance to EGFR-TKIs require further study.

**Methods:**

Targeted sequencing was used for the detection of genomic alterations among patients in our center. Regular cell culture and transfection with plasmids were used to establish NSCLC cell lines over-expressing MDM2 and vector control. We used the MTT assays to calculate the inhibition rate after exposure to erlotinib. Available datasets were used to determine the role of MDM2 in the prognosis of NSCLC.

**Results:**

Four patients harboring concurrent sensitive *EGFR* mutations and *MDM2* amplifications demonstrated insensitivity to EGFR-TKIs in our center. In vitro experiments suggested that *MDM2* amplification induces primary resistance to erlotinib. Over-expressed MDM2 elevated the IC50 value of erlotinib in HCC2279 line and reduced the inhibition rate. In addition, *MDM2* amplification predicted a poor prognosis in NSCLC patients and was associated with a short PFS in those treated with EGFR-TKIs. The ERBB2 pathway was identified as a potential pathway activated by *MDM2* amplification could be the focus of further research.

**Conclusion:**

*MDM2* amplification induces the primary resistance to EGFR-TKIs and predicts poor prognosis in NSCLC patients. MDM2 may serve as a novel biomarker and treatment target for NSCLC. Further studies are needed to confirm the mechanism by which amplified MDM2 leads to primary resistance to EGFR-TKIs.

## Introduction

Lung cancer ranks first among all malignancies in cancer-related mortality, and the 5-year overall survival (OS) is lower than 20% in China (Allemani et al. 2018). Non-small-cell lung cancer (NSCLC) consists of nearly 85% of lung cancer cases (Hou et al. 2019a) and targeted therapeutics based on driver mutations of NSCLC, such as mutations of epidermal growth factor receptor (EGFR) (Santoni-Rugiu et al. 2019) and anaplastic lymphoma kinase (ALK) (Golding et al. 2018), have significantly prolonged the survival of patients. Approximately 50% of Asian NSCLC patients harbor EGFR mutations, while 11–16% of patients in Western countries (Recondo et al. 2018) benefit from treatment with first-generation EGFR-TKIs. Mutations were detected in exons 18 to 21 of EGFR, while the majority of EGFR mutations are exon 19 deletions and exon 21 substitutions of leucine for arginine (L858R) (Recondo et al. 2018; Castellanos et al. 2017). First-generation EGFR-TKIs, including gefitinib and erlotinib, have benefited NSCLC patients, especially Asian patients. According to the IRESSA Pan-Asia Study, patients treated with gefitinib demonstrated longer progression-free survival (PFS) than those treated with standard chemotherapy regimens, including carboplatin and paclitaxel (9.5 months versus 6.3 months) (Mok et al. 2009).

Unfortunately, patients may develop resistance to first generation EGFR-TKIs, which leads to treatment failure. In addition to acquired resistance, multiple genomic alterations have been proven to be associated with primary resistance to EGFR-TKIs, such as the pre-existing T790M mutation (Inukai et al. 2006; Lee et al. 2014), *insulin-like growth factor 1 receptor* (*IGF1R*) mutation (Sharma et al. 2010), *MET* amplification (Turke et al. 2010), *hepatocyte growth factor* (*HGF*) mutation (Yano et al. 2008) and mutations leading to sustained activated signaling in other pathways, including the PI3K/AKT pathway (Tan et al. 2015). In our previous review, we identified the potential relationship between *murine double minute 2* (*MDM2*) amplification and primary resistance to EGFR-TKIs. *MDM2* amplification may activate the bypass signaling pathways, inhibit tumor cell apoptosis, promote the epithelial to mesenchymal transition (EMT) process and tumor angiogenesis and contribute to primary resistance to EGFR-TKIs (Hou et al. 2019b). Therefore, we performed this study to confirm our hypothesis that *MDM2* amplification contributes to the primary resistance to first-generation EGFR-TKIs in NSCLC.

## Methods

### Clinical cases and targeted sequencing

Patients with advanced NSCLC (stage IIIB to IV) seen at our center from July 2015 to March 2018 were selected for targeted sequencing with the patients’ consent (*n* = 141). The detailed sequencing procedure has been described in our previous study (Hou et al. 2018a). Patients harboring concurrent *EGFR* sensitive mutations and *MDM2* amplification were included in this study. All patients were treated with first-generation EGFR-TKIs selected by the patient. The disease evaluation followed Response Evaluation Criteria in Solid Tumors 1.1 (RECIST 1.1). The research was admitted by the Ethics Committee of the Affiliated Hospital of Qingdao University, and the investigations all followed the rules of the Declaration of Helsinki. Written informed consent was signed by all patients when the research began, and all experiments were carried out following the guidelines of the National Health and Family Planning Commission of the PRC.

### Cell lines and cell culture

NSCLC (adenocarcinoma) cell lines were purchased from the cell bank from the Chinese Academy of Sciences (Shanghai, China). *EGFR* mutations were verified in these cell lines (Gandhi et al. 2009; Li et al. 2007). The cell lines were cultured in Roswell Park Memorial Institute (RPMI)-1640 medium with 20% fetal bovine serum (FBS) as well as 1% P/S (100 IU/ml penicillin and 100 IU/ml streptomycin) in a 37 °C humidified atmosphere with 5% CO2. All cell lines were tested for mycoplasma and chlamydia, and all subsequent experiments were used the selected cell lines within six generations.

### Transfection

The cell line was divided into two groups and transfected with plasmids expressing MDM2 (LVRU6GP-MDM2, Fulengene, Guangzhou, China) or empty vector (LVRU6GP-Vector, Fulengene, Guangzhou, China) using Lipofectamine 2000 (Invitrogen, Carlsbad, CA). The transfection process lasted for 48 h, and then the cells were harvested for subsequent experiments.

### RNA extraction and quantitative real-time PCR (qPCR)

TRIzol (Invitrogen, Carlsbad, CA) was used to extract the total RNA from the cultured cells. PrimeScript™ RT Kit (TaKaRa, Otsu, Japan) was used to perform the cDNA synthesis. The qPCR was performed by an FTC-3000p Realtime PCR system (Funglyn Biotech, Shanghai, China) using SYBR Premix EX Taq™ (TaKaRa, Otsu, Japan). The expression levels of RNA were determined by the comparative 2^−ΔΔCT^ method as described in the previous work of our lab (Wang et al. 2015). The PCR primers used in this study are listed in Table S1.

### Western blotting

Cell lysates were centrifuged at 12,000 g for 20 min at 4 °C, and the BCA protein assay reagent kit (Beyotime, Shanghai, China) was used to determine the protein concentrations of the supernatants. The supernatants were mixed with 5 × SDS loading buffer and heated at 95 °C for 5 min. Twenty milligrams of total protein from each sample was separated by SDS-PAGE and transferred to 0.22-μm nitrocellulose (NC) membranes. The membranes were blocked with 5% nonfat dry milk in TBST for 2 h and incubated overnight with the primary antibody. After being washed three times for 30 min with TBST, the membrane was incubated with HRP conjugated secondary antibodies for 2 h at room temperature. We used the ECL reagent (Pierce, Rockford, IL, USA) to visualize the immunoreactive blots. The information of antibody information was concluded in Table S1.

### MTT assay

Transfected cells were seeded (5000 cells/well) in 96-well plates overnight and exposed to proportionally diluted erlotinib HCl (OSI-744) purchased from Selleck ranging from 1 μM to 128 μM. After 24 h incubation, 20 ml of MTT solution (5 mg/ml) was added to the medium, and the cells were incubated at 37 °C for another 4 h. Then we discarded the culture medium and added 150 ml of DMSO to each well. Absorbance (A) was measured at 570 nm using an ELISA plate reader, with background subtraction measurements done at 630 nm. The inhibition rate was calculated as described in our previous study (Hou et al. 2018b): Inhibition rate = 1-[(A570-A630) of treated cells/(A570-A630) of control cells].

### Bioinformatic analyses

Survival analyses were performed online with cBioportal for Cancer Genomics and GEPIA (Tang et al. 2017). In addition, we divided lung adenocarcinoma (LUAD) patients from The Cancer Genome Atlas (TCGA) into two groups, MDM2-high (MDM2-H) and MDM2-low (MDM2-L), and performed Gene Set Enrichment Analysis (GSEA) to acquire the altered signaling pathways between the two groups of patients. (Patients information available at: http://xena.ucsc.edu).

### Statistical analyses

All figures and statistical results in our study were generated by GraphPad Prisma 8.0 software and CorelDRAW 2019. Survival curves were plotted using the Kaplan–Meier method and compared using the log-rank test. *P* values were two-tailed examined for all tests, and *P* < 0.05 was used to define statistical significance.

## Results

### Case reports

**Fig. 1:**
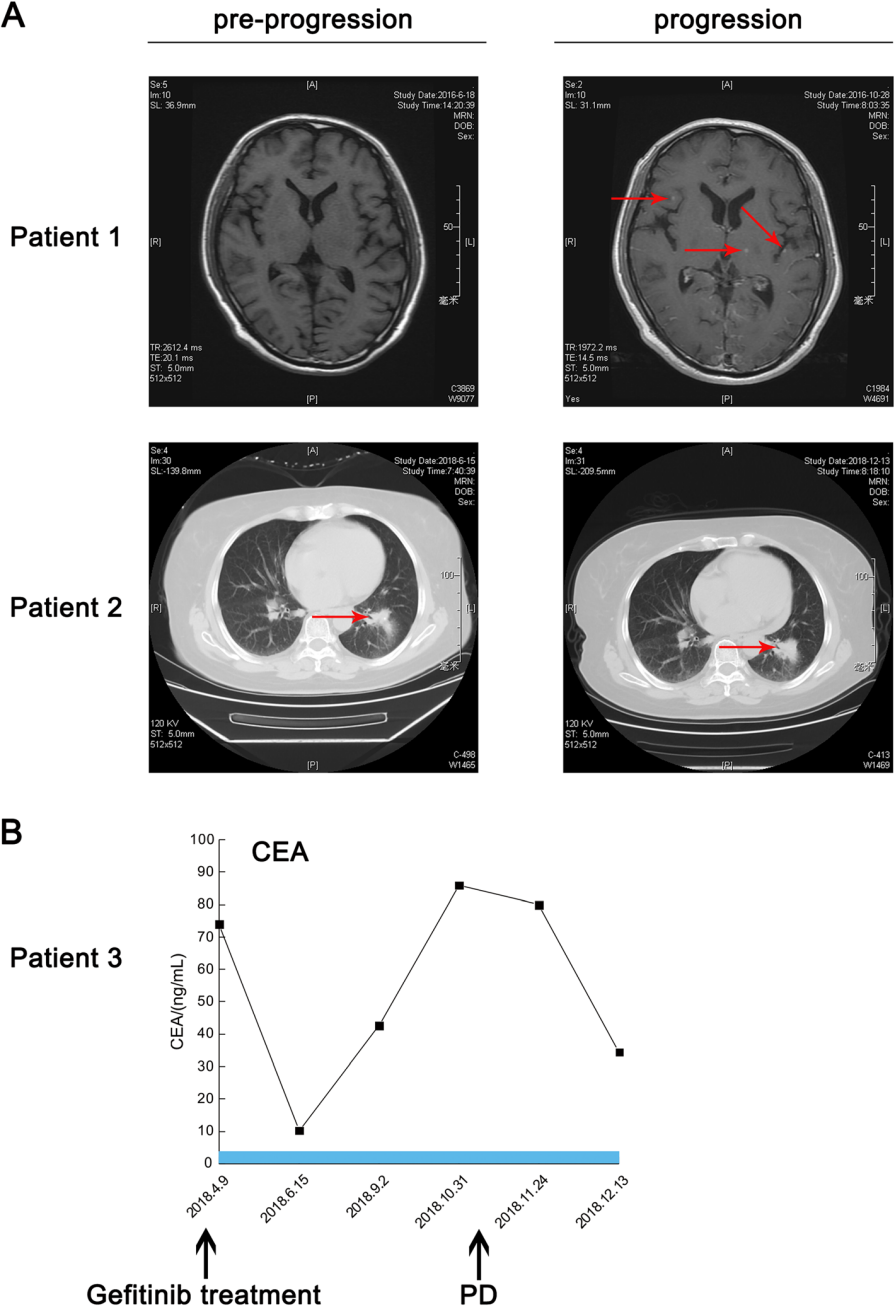
Basic information for selected patients harboring concurrent alterations of *EGFR* sensitive mutations and *MDM2* amplification. **a** CT and MRI scans of patients before and after the progression of the disease: new lesions in central nervous system of patient 1 were detected; the primary lesion of patient 2 was evaluated as progressive disease according to RECIST 1.1; **b** the carcinoembryonic antigen (CEA) change of patient 3 during the treatment process: CEA of patient 3 elevated drastically after approximately 2 months of gefitinib treatment

In total, 4 patients with stage IIIB to IV NSCLC were reported in this study. Targeted sequencing for these patients revealed that all 4 patients harbored concurrent *MDM2* amplification and sensitive *EGFR* mutations, either deletions of exon 19 or the L858R mutation of exon 21. Patients all selected gefitinib (250 mg, qd) as their first-line treatment given the genomic alterations of *EGFR*. However, all patients did not reach the promised PFS values in clinical trials including Asian patients, the mean PFS (mPFS) was 6.25 months, ranging from 5.1 months to 8.1 months, and progressive disease was detected in all patients through CT or MRI scans. Patient 1 was a 62-year-old man diagnosed with stage IV LUAD on May 16, 2016, whose disease progressed after 5.2 months of gefitinib treatment. Multiple brain metastases were found by MRI scan, as shown in Fig. 1a. Patient 2, patient 3 and patient 4 had PFS values of 6.9 months, 8.1 months and 5.1 months, respectively. The maximum diameter of the tumor increased by 20% over the baseline level, and tumor markers were elevated. The change in carcinoembryonic antigen (CEA) level over time in patient 3 is shown in Fig. 1b, and CT scans for patient 2 are shown in Fig. 1a as well. Initial targeted sequencing of the four patients revealed that all patients harbored *MDM2* amplification, therefore, we hypothesized that *MDM2* amplification may induce primary resistance to EGFR-TKIs and result in a poor prognosis of NSCLC patients harboring sensitive *EGFR* mutations. The results of targeted sequencing and detailed information of the four patients are listed in Table 1.

**Table 1:** Basic characteristics and NGS examination of four patients

	Patient 1	Patient 2	Patient 3	Patient 4
**Age**	62	60	59	67
**Gender**	male	female	female	male
**Race**	Asian	Asian	Asian	Asian
**TNM stage**	IV	IV	IV	IIIB
**Pathology**	Moderately differentiated adenocarcinoma	Invasive adenocarcinoma	Poorly differentiated adenocarcinoma	Invasive adenocarcinoma
**EGFR-TKIs**	Gefitinib	Gefitinib	Gefitinib	Gefitinib
**Regimen**	250 mg, qd	250 mg, qd	250 mg, qd	250 mg, qd
**PFS (months)**	5.2	6.9	8.1	5.1
**Genomic alterations**	**EGFR Exon 21 L858R** **MDM2 Amplification** TP53 D208G ERBB4 L770V BLM D997N	**EGFR Exon 19 Deletion** **MDM2 Amplification** CDK4 Amplification ARID1B Q1312K NKX2–1 G115S PTCH1 G1136 RET R475W	**EGFR Exon 19 Deletion** **MDM2 Amplification** EGFR Amplification CDK4 Amplification CTIF R77L APC L1564	**EGFR Exon 21 L858R** **MDM2 Amplification** FLT3 P336T FYN L411M FANCL M89I NCOR1 P347T

### *MDM2* amplification induces primary resistance to EGFR-TKIs

**Fig. 2:**
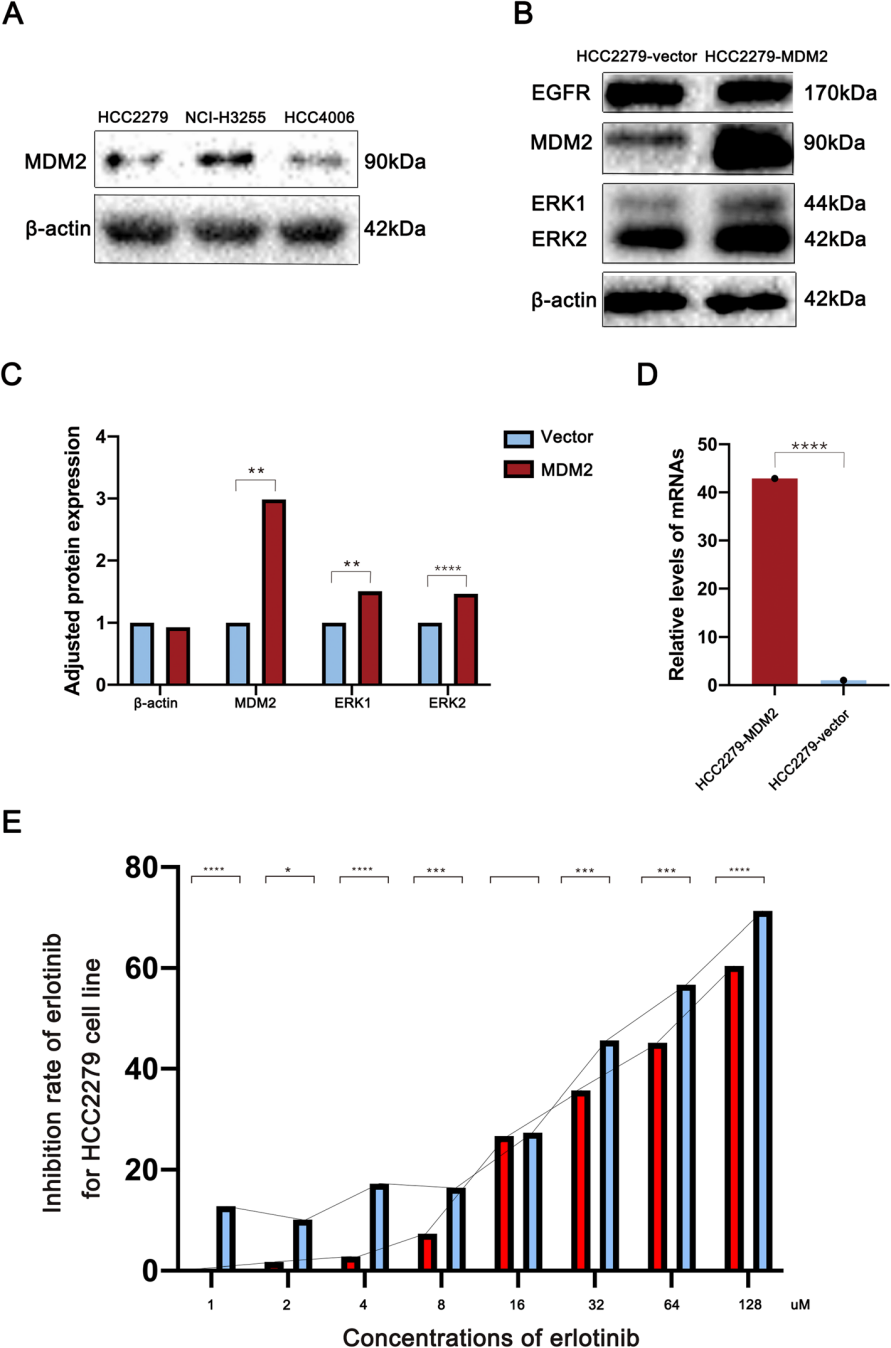
*MDM2* amplification induces the primary resistance to erlotinib. **a** Western blotting results for the evaluation of MDM2 expression level among NSCLC cell lines: the HCC2279 cell line was selected for the subsequent experiments for the medium expression of MDM2; **b** Western blotting results for the target proteins in HCC2279 cell line: β-actin is the protein encoded by the house-keeping gene and balances the protein concentrations between 2 groups; MDM2 expression was upregulated after the transfection of the plasmids; EGFR expression demonstrates no significant difference between 2 groups; ERK proteins including ERK1 and ERK2 were all upregulated in HCC2279-MDM2 group; **c** Quantified results for western blotting; **d** The verification of transfection in HCC2279 cell line: the transcription level of *MDM2* gene was highly upregulated in HCC2279-MDM2 group; **e** The curve of the inhibition rate after exposure to erlotinib with different concentration through the examination of MTT assays in the *MDM2* amplification group and vector control group of HCC2279 cell line: 10uM erlotinib was set as the medium concentration in this experiment, dramatic resistance to erlotinib could be found in HCC2279-MDM2 group in 1uM, 2uM, 4uM, 8uM, 32uM, 64uM and 128uM erlotinib. The inhibition rates were measured via the formula as follow: Inhibition rate = 1-[(A570-A630) of treated cells/(A570-A630) of control cells]

We estimated the expression of MDM2 in three NSCLC cell lines harboring *EGFR* sensitive mutations, including HCC2279, NCI-H3255 and HCC4006 cell lines and HCC2279 cell line were eventually selected for subsequent experiments. The western blotting results for the evaluation of MDM2 expression are shown in Fig. 2a. After transfection with plasmids, we harvested the cells and estimated the relative RNA expression levels of MDM2 between the two groups in HCC2279 cell line. Cells transfected with plasmids expressing MDM2 demonstrated higher expression levels than the vector control group, as shown in Fig. 2d.

After exposure to erlotinib, we compared the inhibition rate of the MDM2 group and vector group in the HCC2279 cell line, as shown in Fig. 2e. As shown in Fig. 2e, the MDM2 group demonstrated the ability to induce primary resistance to EGFR-TKIs, represented by significantly low inhibition rates while exposed to different doses of erlotinib (1 μM, 2 μM, 4 μM, 8 μM, 32 μM, 64 μM and 128 μM). According to previous studies (Li et al. 2007; Yoshida et al. 2015), 10 μM erlotinib inhibits the Tyr845 (Src-dependent phosphorylation) and Tyr1068 (autophosphorylation) phosphorylation of EGFR. Therefore, the MDM2 group demonstrated significant resistance to erlotinib compared with the vector group in the HCC2279 cell line before and after the inhibition of EGFR phosphorylation by erlotinib. In addition, MDM2 amplification significantly elevated the IC50 values of erlotinib in the HCC2279 cell line from 36.5 μM (43.4 μM - 79.6 μM) to 57.6 μM (24.1 μM - 58.7 μM).

The western blotting results in Fig. 2b verified the transfection process and the expression level of target proteins. MDM2 expression was significantly elevated by transfection. In addition, we examined the expression levels of EGFR, ERK1 and ERK2. The expression of EGFR showed no significant change after transfection. However, ERK1 and ERK2 showed a positive correlation with the elevation of MDM2 expression. The quantified results of western blotting are shown in Fig. 2c.

### *MDM2* amplification predicts a poor prognosis in NSCLC

**Fig. 3:**
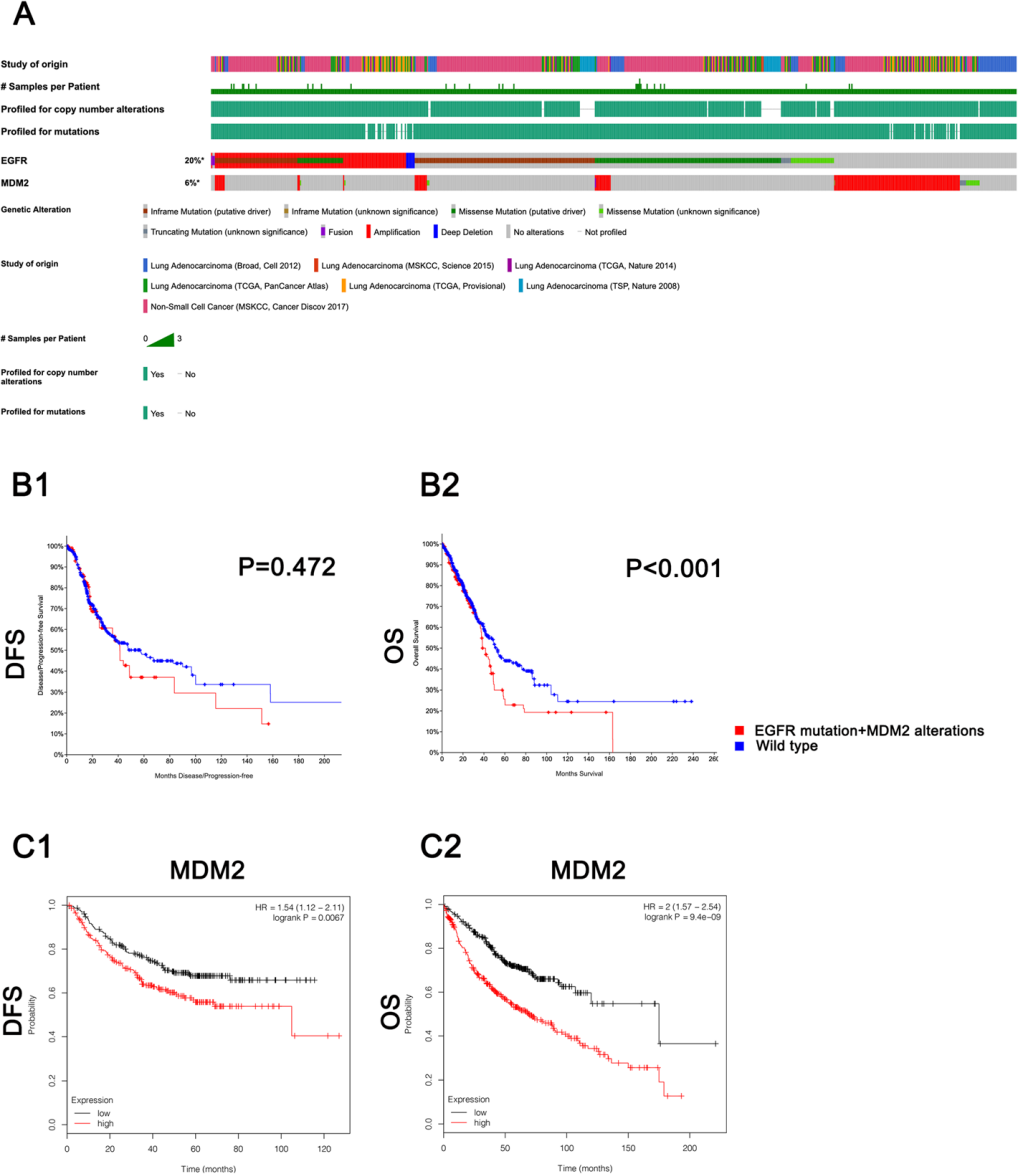
*MDM2* amplification predicts a poor prognosis in NSCLC patients. **a** The genomic signatures of NSCLC patients derived from cBioportal for Cancer Genomics: 20% NSCLC patients were detected harboring *EGFR* alterations and 6% of them were detected harboring *MDM2* alterations; B1-B2. The survival analyses of NSCLC patients grouped by genomic signatures of *EGFR* and *MDM2* (DFS: disease free survival; OS: overall survival): NSCLC patients harboring the concurrent alterations of *EGFR* and *MDM2* have a poor OS (*P* < 0.001); C1-C2. The survival analyses of NSCLC patients grouped by MDM2 expression: NSCLC patients with higher expression of MDM2 demonstrate poor DFS (*P* = 0.0067) and OS (*P* < 0.0001)

*MDM2* amplification induces resistance to EGFR-TKIs in NSCLC cell lines, and we further researched the relationship between *MDM2* amplification and the prognosis in NSCLC. As shown in Fig. 3a, 20% of LUAD patients harbored genomic alterations in *EGFR*, and 6% of LUAD patients harbored genomic alterations in *MDM2*. *MDM2* amplification is the major alteration and accounts for 85.98% of all genomic alterations of *MDM2*. In addition, 1.42% of LUAD patients harbored concurrent *EGFR* mutations and *MDM2* alterations. As shown in Fig. 3 B1, the concurrent genomic alteration of *EGFR* and *MDM2* demonstrated weak ability in predicting the disease free survival (DFS) of LUAD patients (the survival curve of DFS may discriminate the patients but no statistical significance was found) but patients harboring concurrent genomic alterations of *EGFR* and *MDM2* demonstrated poor OS (*P* < 0.001) according to the survival curves shown in Fig. 3 B2. In fact, MDM2 expression itself is a biomarker for predicting the poor DFS (*P* = 0.007) and OS (*P* < 0.001) in NSCLC, as shown in Fig. 3 C1 and C2. The mean DFS in the MDM2-L group was 38.13 months versus 22 months in the MDM2-H group, and the mean OS was 175 months in the MDM2-L group versus 68.67 months in the MDM2-H group.

### MDM2 serves as a potential regulator by affecting EGFR-TKI resistance pathways

To determine the potential pathway altered by MDM2 expression and provide more information for further research, we performed GSEA based on data from the TCGA database, as shown in Figure S1A1 to Figure S1A4. These results revealed that MDM2 may activate the ERBB2 pathway and induce primary resistance to EGFR-TKIs via this pathway. In GSEA, the expression of PI3K (nominal *P* value = 0.018) and SCH1 (nominal *P* value = 0.034), members of the ERBB2 signaling pathway, was up-regulated, and cell motility regulated by ERBB2 (nominal *P* value = 0.020) was activated in the MDM2-H group. In addition, the platelet-derived endothelial growth factor (PDGF)-ERK pathway, which also serves as one of the EGFR-TKI resistance pathways, was also activated in the MDM2-H group (nominal *P* value = 0.002). MDM2, which is a ubiquitinase of P53, is associated with the ubiquitination of the molecules shown in Fig. S1B through the detection of gene-cloud of biotechnology information (GCBI), including MET and IGF1R, which are tightly associated with the resistance to EGFR-TKIs described above. The protein-protein interaction (PPI) plot is shown in Fig. S1C. We further performed enrichment analysis based on these genes through Metascape (an online tool for enrichment analyses), as shown in Fig. S1D. These genes were closely related to pathways inducing EGFR-TKI resistance, including positive regulation of kinase activity (GO: 0033674) and activation of the EGFR-TKI resistance pathway (hsa01521).

## Discussion

EGFR-TKIs benefit NSCLC patients harboring sensitive *EGFR* mutations and prolong survival. However, 20–30% of NSCLC patients harboring sensitive EGFR mutations exhibit primary resistance to EGFR-TKIs (Xu et al. 2016; Zhang et al. 2019). Further research is needed to investigate the mechanisms of primary resistance to EGFR-TKIs. As far as we are concerned, our study is the first to confirm the relationship between *MDM2* amplification and resistance to first-generation EGFR-TKIs. Four NSCLC patients in our study harboring *EGFR* sensitive mutations and *MDM2* amplification had significantly shortened PFS, which drew our attention towards the initial targeted sequencing done before EGFR-TKI treatment. According to the survival analyses based on data from the TCGA database, MDM2 predicts poor prognosis in NSCLC patients, especially a short OS in patients harboring concurrent EGFR mutations and MDM2 alterations. MDM2, which serves as the biological negative regulator of P53, may be a potential target for further NSCLC treatment.

In vitro data in our study suggested that HCC2279 cells over-expressing MDM2 demonstrated the ability to develop primary resistance to EGFR-TKIs regardless of the EGFR phosphorylation. MDM2 overexpression confers resistance to EGFR-TKIs in NSCLC cell lines, and low expression of MDM2 leads to sensitivity to EGFR-TKIs. In this study, the MDM2-overexpressing cell line elevated the IC50 value of EGFR-TKIs and significantly reduced the inhibition rate. This phenomenon led us to investigate the potential pathways altered by MDM2 expression that may be associated with primary resistance to EGFR-TKIs. *ERBB2* amplification was proven to be closely associated with resistance to EGFR-TKIs (Takezawa et al. 2012; Kim et al. 2019). In vitro experiments revealed that ERBB2-overexpressing cell lines were resistant to erlotinib. In addition, *ERBB2* amplification was detected in human samples that demonstrated resistance to EGFR-TKIs. Through GSEA based on MDM2 expression, we found that the ERBB2 signaling pathway was significantly activated in the MDM2-H group, which indicated that MDM2 amplification may induce primary resistance to EGFR-TKIs via the ERBB2 signaling pathway. In addition to effects on the ERBB2 signaling pathway, PDGF was significantly up-regulated in the MDM2-H group. In our previous review (Hou et al. 2019b), MDM2 overexpression up-regulated the expression of PDGF and vascular endothelial growth factor (VEGF), which contributed to sustained cancerous angiogenesis and induced resistance to EGFR-TKIs. Therefore, *MDM2* amplification activates multiple pathways and enables tumor cells to develop resistance to EGFR-TKIs. Admittedly, some limitations exist in our study, and the pathways activated by *MDM2* amplification that contribute to resistance to EGFR-TKIs remain to be further studied.

Several studies support our perspective. Kim et al. (Paez et al. 2004) confirmed that advanced NSCLC patients harboring sensitive *EGFR* mutations and *MDM2* amplification had a shorter PFS during EGFR-TKIs treatment than patients without *MDM2* amplification (6.6 versus 10.4 months; *P* = 0.025). Importantly, *MDM2* amplification is one of the most frequent concurrent alterations in NSCLC patients harboring *EGFR* mutations (Yu et al. 2018). MDM2 may serve as a novel target for NSCLC treatment. Another study proved that the combination treatment of EGFR-TKIs and MDM2 inhibitors can inhibit the proliferation of tumor cells and enhance the anti-tumor effect of EGFR-TKIs (Bianco et al. 2004). In addition to resistance to EGFR-TKIs, *MDM2* amplification was also confirmed to be associated with the insensitivity to radiotherapy (Feng et al. 2016; Koom et al. 2012) and the hyperprogressive disease (HPD) associated with cancer immunotherapy (Kato et al. 2017). In conclusion, MDM2 amplification is related to resistance to multiple cancer therapeutics and may serve as the novel biomarker and treatment target in the future.

## Conclusion

*MDM2* amplification induces resistance to first-generation EGFR-TKIs in NSCLC patients harboring sensitive EGFR mutations. In vitro experiments suggested that MDM2 overexpression endowed HCC2279 cells with primary resistance to erlotinib, as represented by an elevated IC50 value and the reduced inhibition rate. According to GSEA based on MDM2 expression, the ERBB2 and tumor angiogenesis pathways activated by *MDM2* amplification may serve as important pathways inducing primary resistance to EGFR-TKIs. The pathways activated by *MDM2* amplification require further study.

## Supplementary information

**Additional file 1: Table S1.** Antibodies and primer sequences.

**Additional file 2: Figure S1.** The potential pathway activated by MDM2 amplification. A1-A4. The GSEA analyses of NSCLC patients based on MDM2 expression; B. The molecules ubiquitinated by MDM2; C. The PPI plot of molecules ubiquitinated by MDM2; D. The enrichment analyses of molecules ubiquitinated by MDM2.

## Data Availability

All data generated during this study are included in this published article. The datasets generated in the current study are available in the TCGA database and cBioportal for Cancer Genomics.

## Abbreviations

OS: Overall survival
PFS: Progression-free survival
NSCLC: Non-small-cell lung cancer
EGFR: Epidermal growth factor receptor
ALK: Anaplastic lymphoma kinase
IGF1R: Insulin-like growth factor 1 receptor
HGF: Hepatocyte growth factor
MDM2: Murine double minute 2
EMT: Epithelial to mesenchymal transition
LUAD: Lung adenocarcinoma
CEA: Carcinoembryonic antigen
PDGF: Platelet-derived endothelial growth factor
VEGF: Vascular endothelial growth factor
