# Supplementary material for: Primary resistance to first-generation EGFR-TKIs induced by MDM2 amplification in NSCLC
Source: Mol Med. 2020 Jul 1;26:66. doi: 10.1186/s10020-020-00193-z (PMC7329552; doi:10.1186/s10020-020-00193-z)
Supplement: Supplementary file 1 — Additional file 1: Table S1. Antibodies and primer sequences. [file 10020_2020_193_MOESM1_ESM.doc]

**Table S1. Antibodies and primer sequences**

| **Antibodies** |  | |
| --- | --- | --- |
| **Name** | **Manufacturer** | **Number** |
| MDM2 | Cell Signaling Technology | 86934 |
| EGFR | Cell Signaling Technology | 4267 |
| ERK | Cell Signaling Technology | 4695 |
| β-actin | Sigma-Aldrich | A3854 |
| **Primer sequences (5’-3’)** |  | |
| MDM2 | F: CTGAAGAGGGCTTTGATGTT R: AGAAGTTGATGGCTGAGAATAG | |
| GAPDH | F: TGACTTCAACAGCGACACCCA R: CACCCTGTTGCTGTAGCCAAA | |
