# Supplementary material for: Primary resistance to first-generation EGFR-TKIs induced by MDM2 amplification in NSCLC
Source: Mol Med. 2020 Jul 1;26:66. doi: 10.1186/s10020-020-00193-z (PMC7329552; doi:10.1186/s10020-020-00193-z)
Supplement: Supplementary file 2 — Additional file 2: Figure S1. The potential pathway activated by MDM2 amplification. A1-A4. The GSEA analyses of NSCLC patients based on MDM2 expression; B. The molecules ubiquitinated by MDM2; C. The PPI plot of molecules ubiquitinated by MDM2; D. The enrichment analyses of molecules ubiquitinated by MDM2. [file 10020_2020_193_MOESM2_ESM.docx]

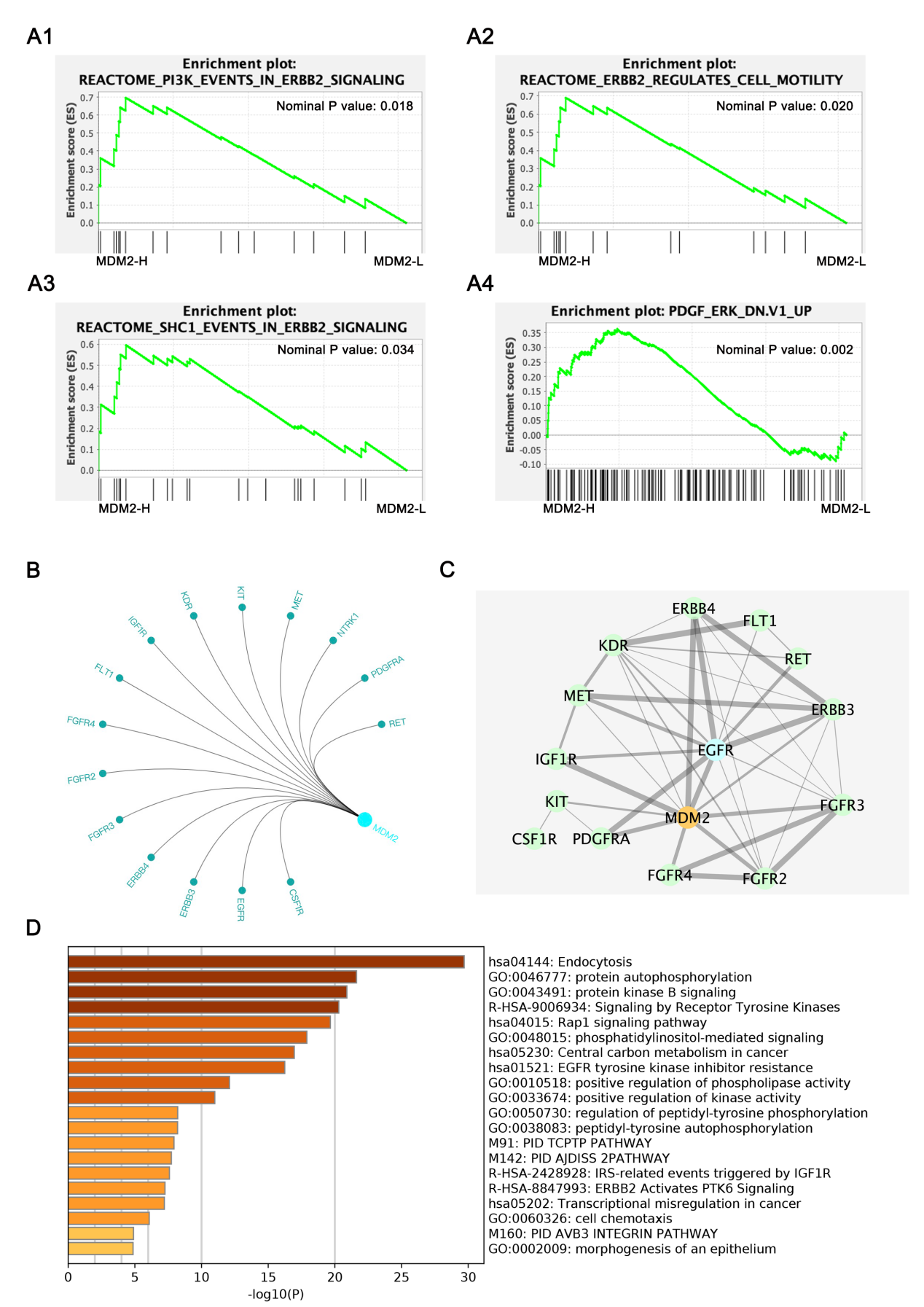


Figure S1. The potential pathway activated by *MDM2* amplification. A1-A4. The GSEA analyses of NSCLC patients based on MDM2 expression; B. The molecules ubiquitinated by MDM2; C. The PPI plot of molecules ubiquitinated by MDM2; D. The enrichment analyses of molecules ubiquitinated by MDM2.
